# FGF-23/Vitamin D Axis in Type 1 Diabetes: The Potential Role of Mineral Metabolism in Arterial Stiffness

**DOI:** 10.1371/journal.pone.0140222

**Published:** 2015-10-13

**Authors:** Gemma Llauradó, Ana Megia, Albert Cano, Olga Giménez-Palop, Inmaculada Simón, Montserrat González-Sastre, Eugenio Berlanga, Sonia Fernández-Veledo, Joan Vendrell, José-Miguel González-Clemente

**Affiliations:** 1 Hospital Universitari Joan XXIII de Tarragona, Institut d’Investigacions Sanitàries Pere Virgili (IISPV), Universitat Rovira i Virgili, Tarragona, Spain; 2 Department of Endocrinology and Nutrition, Hospital del Mar, IMIM (Hospital del Mar Medical Research Institute), Barcelona, Spain; 3 Department of Endocrinology and Nutrition, Hospital de Sabadell, Corporació Sanitària i Universitària Parc Taulí (Universitat Autònoma de Barcelona), Sabadell, Spain; 4 Department of Ophthalmology, Hospital de Sabadell, Corporació Sanitària i Universitària Parc Taulí (Universitat Autònoma de Barcelona), Sabadell, Spain; 5 Biochemistry Department, UDIAT, Corporació Sanitària i Universitària Parc Taulí (Universitat Autònoma de Barcelona), Sabadell, Spain; 6 Centro de Investigación Biomédica en Red de Diabetes y Enfermedades Metabólicas Asociadas (CIBERDEM), Instituto de Salud Carlos III, Madrid, Spain; Virgen Macarena University Hospital, School of Medicine, University of Seville, SPAIN

## Abstract

**Objective:**

To investigate the usefulness of Fibroblast Growth Factor 23 (FGF-23) and vitamin D as possible biomarkers of pre-clinical atherosclerosis, assessed as arterial stiffness (AS), in a group of subjects with type 1 diabetes (T1DM) and no previous cardiovascular events.

**Research Design and Methods:**

68 T1DM patients and 68 age- and sex-matched controls were evaluated for 1) age, sex, diabetes duration, physical activity, smoking, alcohol intake, BMI, blood pressure, fasting plasma glucose, HbA_1c_, estimated glomerular filtration rate (eGFR) and lipid profile; 2) microvascular complications; 3) blood concentrations of FGF-23 and mineral metabolism parameters (calcium, phosphate, parathyroid hormone (PTH) and 25-hydroxy-vitamin D (25(OH)D)); 4) AS, assessed as aortic pulse wave velocity (aPWV); and 5) low-grade inflammation (hsCRP, IL-6, sTNFαR1, sTNFαR2) and endothelial dysfunction (ED) markers (ICAM-1, VCAM-1, E-Selectin).

**Results:**

Patients with T1DM had higher aPWV compared with controls (p<0.001), but they did not present differences in 25(OH)D (70.3(50.4–86.2)nmol/L vs. 70.7(59.7–83.0)nmol/L; p = 0.462) and in FGF-23 plasma concentrations (70.1(38.4–151.9)RU/mL vs. 77.6(51.8–113.9)RU/mL; p = 0.329). In T1DM patients, higher concentrations of FGF-23 were positively associated with aPWV after adjusting for eGFR and classical cardiovascular risk factors (model 1: ß = 0.202, p = 0.026), other mineral metabolism parameters (model 2: ß = 0.214, p = 0.015), microvascular complications, low-grade inflammation and ED markers (model 3: ß = 0.170, p = 0.045). Lower 25(OH)D concentrations were also associated with higher aPWV after adjusting for all the above-mentioned factors (model 3: ß = -0.241, p = 0.015).

**Conclusions:**

We conclude that both FGF-23 plasma concentrations (positively) and 25(OH)D serum concentrations (negatively) are associated with AS in patients with T1DM and no previous cardiovascular events.

## Introduction

Cardiovascular disease is the major cause of mortality in type 1 diabetes mellitus (T1DM) [1]. As much as 10% of premature coronary artery disease (CAD) morbidity and mortality in the general population is due to T1DM, but conventional cardiovascular risk factors account for no more than 25% of this excess in cardiovascular risk [2]. Therefore, the pathophysiological mechanisms underlying cardiovascular events in T1DM are not completely understood.

Existing evidence suggests that an abnormal mineral metabolism, and particularly vitamin D deficiency, may promote atherosclerosis. Recently, low blood concentrations of vitamin D have been associated with an increased risk of excess all-cause and cardiovascular mortality in the general population [3], as well as in patients with type 2 diabetes (T2DM) [4] and T1DM [5]. The discovery of Fibroblast Growth Factor 23 (FGF-23) has introduced a new perspective linking vitamin D metabolism and cardiovascular disease.

FGF-23 is a circulating peptide hormone secreted by bone cells, with three main physiological actions. First, it induces phosphaturia through a reduction in the reabsorption of phosphate at the proximal tubules. Second, it lowers circulating 1,25-dihydroxy-vitamin D concentrations through a decrease in the activation and an increase in the degradation of 25-hydroxy-vitamin D (25(OH)D). Third, FGF-23 suppresses the transcription of the parathyroid hormone (PTH) gene and PTH secretion [6]. It acts through the activation of the FGF receptor/Klotho co-receptor complexes. FGF-23 is increased in patients with chronic kidney disease (CKD) [7]. In these patients, it has emerged as one of the most powerful predictors of adverse cardiovascular outcomes and CKD progression across all strata of CKD [8–10]. Recent studies reported that FGF-23 concentrations are associated with cardiovascular events and mortality in two community-based settings [11–13]. The possible mechanisms for an association of FGF-23 with cardiovascular events are largely unclear, but recent data suggest that this hormone may be a marker of inflammation, insulin resistance and visceral fat accumulation [14].

Recently, we demonstrated that patients with T1DM have an increased inflammatory tone associated with arterial stiffness (AS) before clinical atherosclerotic manifestation [15]. The increased risk of cardiovascular disease in patients with T1DM is well documented. However, there is still a need to find new biomarkers to improve cardiovascular risk prediction, especially in those patients in pre-clinical states, prior to the appearance of a first cardiovascular event [16].

Consequently, we hypothesize that the FGF-23/vitamin D axis may be unbalanced in patients with T1DM with early stages of cardiovascular disease. We investigated blood concentrations of FGF-23 and 25(OH)D and their potential relationships with AS and classical cardiovascular risk factors in a group of patients with T1DM and no previous cardiovascular events.

## Materials and Methods

### Study subjects

Sixty-eight patients with T1DM, aged 18–65 years and 68 age- and sex-matched controls were included in the study. Patients with T1DM were consecutively recruited from our outpatient clinic. The control group was recruited from hospital staff members and their relatives and friends. Exclusion criteria included: i) preexisting CKD with renal failure, ii) any other acute/chronic condition associated with an inflammatory response (e.g., acute or chronic inflammatory or infectious diseases), iii) use of anti-inflammatory drugs in the previous 6 months and IV) presence of previous cardiovascular events. The study protocol was approved by our hospital ethics committee (Clinical Research Ethics Committee of Hospital of Sabadell) and conducted in accordance with the Declaration of Helsinki. All subjects gave their written informed consent before participating in the study.

### Study design

All subjects underwent standardized anamnesis and physical examination. The following information was recorded using a predefined standardized form: age, sex, diabetes duration, physical activity (International Physical Activity Questionnaire) [17], smoking, alcohol intake, insulin dose or the use of any other medical treatment, and date of evaluation. Body weight, height, and waist and hip circumferences were registered. Systolic and diastolic blood pressure (SBP and DBP, respectively) were measured and mean arterial pressure (MAP) was calculated as 1/3 SBP + 2/3 DBP. Microvascular complications were evaluated as previously described [15]. All women were evaluated in the follicular phase of the menstrual cycle. After an overnight fast, venous blood samples were taken and complete blood counts, fasting plasma glucose, HbA_1c_, creatinine, lipid profile and mineral metabolism parameters (calcium, phosphate, PTH and 25(OH)D) were determined. In addition, aliquots of plasma and serum were stored at -80°C until processing. From these aliquots, FGF-23, high-sensitivity C-reactive protein (hsCRP), interleukin 6 (IL-6) and soluble fractions of tumor necrosis factor α receptors 1 and 2 (sTNFαR1 and sTNFαR2, respectively), soluble intercellular adhesion molecule-1 (sICAM-1), soluble vascular cell adhesion molecule-1 (sVCAM-1), and E-selectin were determined.

### Laboratory analyses

HbA_1c_ was determined by high-performance liquid chromatography (Menarini Diagnostics, Firenze, Italy). Total serum cholesterol, triglycerides, HDL cholesterol, and LDL cholesterol were measured using standard enzymatic methods. Estimated glomerular filtration rate (eGFR) was calculated using the Chronic Kidney Disease-Epidemiology (CKD-EPI) equation on the basis of serum creatinine, age, sex and race [18]. Serum 25(OH)D was measured using an ELISA kit (BioVendor, Brno, Czech Republic). Plasma FGF-23 was measured using an ELISA kit (Immunotopics Inc. San Clemente, CA, USA). Intra- and inter-assay coefficients of variation were 5% and 7.8% for 25(OH)D and 2.4% and 4.7% for FGF-23, respectively. hsCRP was determined by immunonephelometry (Siemens, Munich, Germany). IL-6 was determined by ELISA (R&D Systems, Oxon, UK), as well as sTNFαR1 (HyCult Biotech, Uden, The Netherlands) and sTNFαR2 (R&D Systems, Oxon, UK). Concentrations of sICAM-1, sVCAM-1 and E-selectin were also determined by ELISA (Wuhan Boster Biological Technology, Ltd., Wuhan, China) as previously described [15,19].

### Assessment of arterial stiffness: aPWV

AS is an early sign of atherosclerosis [20] and predicts cardiovascular events independently of classical cardiovascular risk factors in several populations [21], with aortic pulse wave velocity (aPWV) used as the gold standard for its assessment [22].

The aPWV measurements were performed in accordance with the recommendations of a recent consensus on measuring AS [22]. The method has been previously described in detail [15]. In brief, aPWV was determined by sequential applanation tonometry using a Millar tonometer (SPC-301, Millar Instruments, Houston, TX, USA) at the carotid and femoral arteries, gated to a three-lead electrocardiography (ECG) using the SphygmoCor^®^ system (AtCor Medical Pty Ltd, West Ryde (Sydney), NSW, Australia). Those aPWV recordings not satisfying the automatic quality controls specified by the SphygmoCor^®^ software were rejected. The mean of two aPWV measurements was taken for each subject for all calculations. Data were available for all the participants included in the study.

### Statistical analyses

All data were tested for normality using the Shapiro-Wilk test. Data are presented as percentage, mean (SD) for normally distributed variables, or median (interquartile range) for non-normally distributed variables. The analyses were performed in the whole population after excluding the presence of sex interaction (p for interaction FGF23*sex for aPWV = 0.960). Differences between groups (patients with T1DM vs. controls) were analyzed using the χ^2^test for comparisons of proportions, and the unpaired t-test or the Mann-Whitney U test for comparisons of normally and non-normally distributed quantitative variables, as needed. Pearson’s or Spearman’s correlation coefficients were calculated to analyze the relationship between normally and non-normally distributed variables, as appropriate. To assess the likely association between FGF-23 and AS adjusting for potential confounders (and the other mineral metabolism parameters), several multiple linear regression models were employed, always treating aPWV as the dependent variable. All variables associated in the univariate analyses (p<0.20) and those variables known or likely to be associated with AS (based on previous literature) were included in those regression models as independent variables. Non-normally distributed variables were used after performing a log_10_-transformation. As low-grade inflammation and endothelial dysfunction (ED) markers were measured only once, their possible association (if any) with aPWV and FGF-23 was likely to be underestimated. To address this issue, a Z-score was calculated for each of these markers in each subject, as previously described ((value in the individual minus mean value in the study population) / SD) [15,19]. Subsequently, a value of low-grade inflammation general score was calculated for each subject as: (Z-score of hsCRP + Z-score of IL-6 + Z-score of sTNFαR1 + Z-Score of sTNFαR2)/4. Likewise, an ED general score was calculated as: (Z-score of ICAM-1 + Z-score of VCAM-1 + Z-score of E-selectin)/3. Two-tailed p-values <0.05 were considered statistically significant. The calculations were made using STATA v.13.1 for Mac (StataCorp LP, College Station, TX).

## Results

We evaluated 68 patients with T1DM and 68 age- and sex-matched controls (n = 136). The main clinical and analytical characteristics of the study population are shown in Table 1. Of the 136 subjects, 8 were on antihypertensive drugs (7 patients), 15 were on statins (14 patients) and 6 were on antiplatelet drugs (all with T1DM). None of them were treated with calcium or vitamin D supplements.

**Table 1:** Clinical characteristics of study population.

	Control group (n = 68)	Type 1 diabetes (n = 68)	p
Age (years)	35.4 (10.2)	35.3 (10.1)	0.945
Sex (male) (n, %)	34 (50)	34 (50)	1.000
Current smokers (n, %)	16 (23.5)	24 (35.3)	0.252
Alcohol intake (g/day)	1.43 (0.00–5.36)	1.43 (0.00–5.71)	0.886
Physical activity (METS-min/week)	1386.0 (784.5–2079.0)	1416.0 (713.3–2367.0)	0.791
Family history of CHD (n, %)	6 (8.8)	3 (4.4)	0.493
Family history of T2DM (n, %)	12 (17.6)	16 (23.5)	0.396
Family history of T1DM (n, %)	1 (1.5)	5 (7.4)	0.208
Hypertension (n, %)	3 (4.4)	17 (25.0)	0.001
Dyslipidaemia (n, %)	34 (50)	32 (47.1)	0.732
*Diabetes*			
Diabetes duration (years)	-	13.0 (7.3–19.0)	-
Microvascular complications (n, %)	-	16 (23.5)	-
Retinopathy (n, %)	-	10 (14.7)	-
None (n, %)	-	58 (85.3)	-
Non-proliferative (n, %)	-	6 (8.8)	-
Proliferative (n, %)	-	4 (5.9)	-
Nephropathy (n, %)	-	9 (13.2)	-
Peripheral polyneuropathy (n, %)	-	0 (0)	-
*Body composition*			
BMI (kg/m^2^)	24.0 (3.1)	25.7 (3.6)	0.003
Waist (cm)	83.7 (11.3)	85.3 (12.0)	0.429
WHR	0.85 (0.1)	0.86 (0.1)	0.465
*Blood pressure*			
Systolic blood pressure (mmHg)	120.6 (10.4)	125.0 (12.1)	0.025
Diastolic blood pressure (mmHg)	70.8 (8.4)	72.9 (8.3)	0.154
Mean arterial pressure (mmHg)	87.4 (8.6)	90.3 (8.7)	0.059
*Laboratory parameters*			
Serum creatinine (μmoll/L)	75.2 (13.0)	76.9 (14.0)	0.450
eGFR (ml/min/1.73m^2^)	102.4 (13.3)	100.7 (14.2)	0.467
Fasting plasma glucose (mmol/L)	4.67 (0.53)	9.15 (3.66)	<0.001
HbA_1c_ (%)	5.3 (5.2–5.5)	7.5 (6.8–8.7)	<0.001
HbA_1c_ (mmol/mol)	34 (33–37)	58 (51–72)	<0.001
Urinary ACR (mg/mmol)	0.39 (0.28–0.57)	0.36 (0.24–0.69)	0.716
Total Cholesterol (mmol/L)	5.16 (1.33)	4.80 (0.87)	0.070
Triglycerides (mmol/L)	0.80 (0.62–1.17)	0.78 (0.61–0.97)	0.422
HDL-Cholesterol (mmol/L)	1.50 (1.19–1.87)	1.70 (1.21–1.90)	0.369
LDL-Cholesterol (mmol/L)	2.81 (2.25–3.58)	2.52 (2.14-.11)	0.028
*Mineral Metabolism*			
Calcium (mmol/L)	2.4 (2.3–2.4)	2.3 (2.3–2.4)	<0.001
Phosphate (mmol/L)	3.5 (0.1)	3.4 (0.1)	0.231
PTH (pmol/L)	3.2 (2.5–4.6)	3.3 (2.4–4.5)	0.626
25(OH)D (nmol/L)	70.7 (59.7–83.0)	70.3 (50.4–86.2)	0.462
FGF-23 (RU/mL)	77.6 (51.8–113.9)	70.1 (38.4–151.9)	0.329
*Low grade inflammation and endothelial dysfunction markers*			
hsCRP (mg/L)	0.8 (0.4–1.5)	1.3 (0.5–2.8)	0.038
IL-6 (pg/mL)	0.3 (0.2–0.5)	0.6 (0.3–1.1)	<0.001
sTNFαR1 (pg/mL)	1535.1(1190.0–2845.6)	2592.3(1560.8–3021.3)	0.014
sTNFαR2 (pg/mL)	2201.8(1880.2–2561.5)	2568.9(2153.9–3024.0)	0.001
Low-grade Inflammation score	-0.35 (-0.63–0.16)	0.12 (-0.20–0.55)	<0.001
ICAM-1 (ng/mL)	55.1 (48.3–62.7)	58.1 (51.8–69.0)	0.063
VCAM-1 (ng/mL)	715.8 (541.2–954.7)	782.6 (628.6–1090.2)	0.128
E-Selectin (ng/mL)	73.6 (50.9–99.1)	104.6 (68.5–184.9)	<0.001
Endothelial dysfunction score	-0.28 (-0.58–0.02)	0.14 (-0.27–0.58)	<0.001
*Arterial stiffness*			
aPWV (m/s)	6.1 (5.5–6.7)	6.8 (6.0–7.9)	<0.001

Data are given as percentages, mean (SD) or median (interquartile range). CHD: Coronary heart disease. T2DM: type 2 diabetes. T1DM: type 1 diabetes. BMI: body mass index. WHR: waist-to-hip ratio. eGFR: estimation of glomerular filtration rate. ACR: Urinary albumin to creatinine ratio. PTH: parathyroid hormone. 25(OH)D: 25-hydroxy-vitamin D. FGF-23: Fibroblast growth factor 23. hsCRP: high-sensitivity C-reactive protein. IL-6: interleukin 6. sTNFαR1: soluble fraction of tumor necrosis factor α receptor 1. sTNFαR2: soluble fraction of tumor necrosis factor α receptor 2. ICAM-1: soluble intercellular adhesion molecule-1. VCAM-1: soluble vascular cell adhesion molecule-1. aPWV: aortic pulse wave velocity.

Patients with T1DM were more hypertensive and had higher values of BMI, fasting plasma glucose, HbA_1c_, aPWV, and general scores of low-grade inflammation and ED than controls. Additionally, patients with T1DM presented lower concentrations of LDL-cholesterol, probably due to the higher number of individuals on statins in this group. Patients with T1DM presented lower calcium concentrations compared with controls (Table 1). There were no differences for the other mineral metabolism parameters analyzed between groups (phosphate, PTH and 25(OH)D). The number of subjects with vitamin D deficiency (defined as 25(OH)D below 50 nmol/L) did not differ between groups (17 patients with T1DM vs. 11 controls; p = 0.202). This was also the case for FGF-23 concentrations between groups (70.1 (38.4–151.9) RU/ml vs. 77.6 (51.8–113.9) RU/ml; p = 0.329). If we exclude from the control group, those subjects with the presence of at least one cardiovascular risk factor (such as arterial hypertension or dyslipidemia), the results remained unchanged: patients with T1DM still had higher aPWV values (aPWV: 5.7 (5.4–6.5) vs. 6.8 (6.0–7.9); p = 0.004) and FGF23 concentrations (76.9 (51.6–112.2) vs. 70.1 (38.4–151.9); p = 0.441) and vitamin D concentrations (72.1 (59.7–80.7) vs. 70.3 (50.4–86.2); p = 0.602) remained similar between both groups.

To evaluate the potential association of FGF-23 and other mineral metabolism parameters with aPWV, several multiple linear regression models were performed (Table 2 for the whole population, Table 3 for the controls and Table 4 for patients with T1DM). Models were adjusted successively for: age, sex, eGFR, classical cardiovascular risk factors (tobacco, arterial hypertension, dyslipidemia and BMI) (model 1), mineral metabolism parameters (model 2) and T1DM duration, microvascular complications (just for patients with T1DM), low-grade inflammation and ED markers (model 3). In all models including 25(OH)D concentrations, an additional adjustment for seasonality was taken into account to adjust for potential variations.

**Table 2:** Association of FGF-23 with aPWV in the whole population.

	B	SD	β	95%CI	p
Model 1 (age, sex, eGFR and classical cardiovascular risk factors) (R^2^ = 0.546; p<0.001)					
Age	0.054	0.009	0.383	0.036–0.071	<0.001
BMI	0.153	0.026	0.372	0.001–0.205	<0.001
Hypertension (N/Y)	0.816	0.285	0.187	0.252–1.380	0.005
T1DM (N/Y)	0.396	0.177	0.140	0.046–0.746	0.027
FGF-23	0.001	0.001	0.115	0.000–0.002	0.059
Model 2 (model 1 + mineral metabolism) (R^2^ = 0.573; p<0.001)					
Age	0.053	0.009	0.374	0.035–0.070	<0.001
BMI	0.149	0.026	0.364	0.097–0.200	<0.001
Hypertension (N/Y)	0.796	0.282	0.182	0.239–1.353	0.005
T1DM (N/Y)	0.430	0.179	0.153	0.076–0.784	0.018
25(OH)D	-0.006	0.003	-0.133	-0.012–-0.001	0.036
FGF-23	0.001	0.001	0.115	-0.000–0.002	0.053
Model 3 (model 2 + inflammation and ED) (R^2^ = 0.574; p<0.001)					
Age	0.053	0.009	0.374	0.036–0.071	<0.001
BMI	0.148	0.026	0.364	0096–0.200	<0.001
Hypertension (N/Y)	0.850	0.290	0.182	0.275–1.424	0.004
T1DM (N/Y)	0.415	0.180	0.153	0.059–0.772	0.023
25(OH)D	-0.006	0.003	-0.133	-0.012–-0.001	0.042
FGF-23	0.001	0.001	0.115	-0.000–0.002	0.054

Model 1 adjusted for age, sex (male/female), eGFR, current smoking (No/Yes), arterial hypertension (No/Yes), dyslipidaemia (No/Yes), BMI, TD1M (No/Yes) and FGF-23. Model 2 adjusted for covariates in model 1 and reflecting mineral metabolism (calcium, phosphate, PTH and 25(OH)D). Model 3 adjusted for covariates in model 2 and low-grade inflammation general score and ED score. 25(OH)D were additionally adjusted for seasonality.

**Table 3:** Association of FGF-23 with aPWV in the control group.

	B	SD	β	95%CI	p
Model 1 (age, sex, eGFR and classical cardiovascular risk factors) (R^2^ = 0.540; p<0.001)					
Age	0.058	0.010	0.500	0.037–0.079	<0.001
Hypertension (N/Y)	1.425	0.522	0.250	0.318–2.47	0.008
BMI	0.090	0.036	0.237	0.019–0.162	0.014
FGF-23	-0.001	0.001	-0.051	-0.002–0.001	0.561
Model 2 (model 1 + mineral metabolism) (R^2^ = 0.582; p<0.001)					
Age	0.059	0.011	0.509	0.038–0.080	<0.001
Hypertension (N/Y)	1.370	0.522	0.242	0.326–2.414	0.011
BMI	0.080	0.037	0.214	0.007–0.153	0.033
25(OH)D	-0.004	0.004	-0.106	-0.012–0.003	0.247
FGF-23	-0.001	0.001	-0.068	-0.002–0.001	0.440
Model 3 (model 2 + inflammation and ED) (R^2^ = 0.633; p<0.001)					
Age	0.069	0.010	0.558	0.050–0.882	<0.001
Hypertension (N/Y)	2.561	0.576	0.268	1.406–3.715	<0.001
Sex (M/F)	-0.474	0.231	-0.151	-0.936–-0.012	0.045
25(OH)D	-0.003	0.004	-0.124	-0.011–0.004	0.373
FGF-23	-0.001	0.001	-0.057	-0.002–0.001	0.544

Model 1 adjusted for age, sex (male/female), eGFR, current smoking (No/Yes), arterial hypertension (No/Yes), dyslipidaemia (No/Yes), BMI and FGF-23. Model 2 adjusted for covariates in model 1 and reflecting mineral metabolism (calcium, phosphate, PTH and 25(OH)D). Model 3 adjusted for covariates in model 2 and low-grade inflammation general score and ED score. 25(OH)D were additionally adjusted for seasonality.

**Table 4:** Association of FGF-23 with aPWV in the patients with T1DM.

	B	SD	β	95%CI	p
Model 1 (age, sex, eGFR and classical cardiovascular risk factors) (R^2^ = 0.529; p<0.001)					
Age	0.049	0.014	0.328	0.021–0.079	0.001
BMI	0.180	0.038	0.427	0.104–0.257	<0.001
FGF-23	0.002	0.001	0.202	0.001–0.003	0.026
Hypertension (N/Y)	0.738	0.366	0.192	0.007–1.469	0.048
Model 2 (model 1 + mineral metabolism) (R^2^ = 0.580; p<0.001)					
Age	0.043	0.014	0.282	0.015–0.071	0.004
BMI	0.183	0.037	0.432	0.109–0.257	<0.001
FGF-23	0.02	0.001	0.214	0.001–0.003	0.015
25(OH)D	-0.011	0.005	-0.234	-0.020–-0.002	0.018
Hypertension(N/Y)	0.771	0.351	0.200	0.069–1.473	0.032
Model 3 (model 2 + duration and microvascular complications + inflammation and ED) (R^2^ = 0.587; p<0.001)					
Age	0.054	0.013	0.353	0.027–0.080	<0.001
BMI	0.182	0.037	0.431	0.109–0.255	<0.001
Microvascular complications (N/Y)	0.783	0.319	0.203	0.144–1.422	0.017
FGF-23	0.001	0.002	0.170	0.001–0.003	0.045
25(OH)D	-0.011	0.005	-0.241	-0.020–-0.002	0.015

Model 1 adjusted for age, sex, eGFR, current smoking (No/Yes), arterial hypertension (No/Yes), dyslipidaemia (No/Yes), BMI and FGF-23. Model 2 adjusted for covariates in model 1 and variables reflecting mineral metabolism (calcium, phosphate, PTH and 25(OH)D). Model 3 adjusted for covariates in model 2 and duration of diabetes (years) and the presence of microvascular complications (diabetic retinopathy, nephropathy and peripheral polyneuropathy) and low-grade inflammation general score and endothelial dysfunction score. 25(OH)D were additionally adjusted for seasonality.

In the whole population, and after adjusting for all the potential confounders, higher FGF-23 plasma concentrations showed a tendency to be positively associated with aPWV, but without reaching statistical significance (model 3: ß = 0.115, p = 0.054). By contrast, serum concentrations of 25(OH)D were negatively associated with aPWV (model 3: ß = -0.133, p = 0.042) (Table 2). In the group of controls, neither FGF-23 nor 25(OH)D were significantly associated with aPWV (Table 3). In patients with T1DM, higher concentrations of FGF-23 were positively associated with aPWV, even after adjusting for eGFR and classical cardiovascular risk factors (model 1: ß = 0.202, p = 0.026), other mineral metabolism parameters (model 2: ß = 0.214, p = 0.015), T1DM duration, microvascular complications, low-grade inflammation and ED (model 3: ß = 0.170, p = 0.045). Lower serum concentrations of 25(OH)D were also associated with an increase in aPWV when mineral metabolism parameters were taken into account (model 2: ß = -0.238, p = 0.018) and still remained significant in the final model (model 3: ß = -0.241, p = 0.015).

## Conclusions

In this study we highlight FGF-23 as a potential biomarker associated with increased AS in patients with T1DM without previous cardiovascular events. Furthermore, serum vitamin D concentrations were inversely associated with AS in these patients.

FGF-23 has been revealed as a potential useful biomarker not only for cardiovascular disease in high-risk populations, such as CDK patients [8–10], but even in the general population [11–13]. In the current study, we explored the usefulness of this peptide in combination with its counter-regulatory hormone, vitamin D, in association with established subtle markers of cardiovascular disease in T1DM. Despite similar FGF-23 concentrations in T1DM and controls, higher FGF-23 concentrations were associated with higher aPWV values in T1DM, even after adjusting for potential confounders. This is the first time that this peptide has been explored in T1DM, linking circulating levels with a pre-clinical measurement of atherosclerosis such as AS. There is only a previous study evaluating FGF-23 in diabetes. That study was performed in patients with T2DM and the authors, in agreement with our data, found similar concentrations of FGF-23 in patients with T2DM and in controls. Interestingly, the study describes a positive association between FGF-23 concentrations and the intima-media thickness, another validated measurement of pre-clinical atherosclerosis [23]. Notwithstanding the absence of causality in the associative observational studies, all of the above-mentioned data support a potential role of FGF-23 in the initial phases involved in cardiovascular disease.

The mechanisms underlying FGF-23 bioactivity in vivo are unclear although some authors have suggested that FGF-23 may directly contribute to glomerular damage and enhanced proteinuria, as the link between FGF-23 and poor cardiovascular outcomes [8,24,25]. In our study, the association between FGF-23 and AS remained significant even after adjustments for eGFR (renal function) and the presence of microvascular complications (including diabetic nephropathy). This finding agrees with the results of previous studies in general populations [11–13], suggesting that FGF-23 may provide new insights into cardiovascular disease beyond renal function and the presence of albuminuria.

Several hypotheses have been proposed to explain the link between FGF-23 and cardiovascular disease. FGF-23 could indirectly modify cardiovascular risk by regulating essential parameters of mineral metabolism, such as serum phosphate, calcium, PTH and vitamin D [26]. However, in our study, FGF-23 was still associated with AS after adjusting for all these mineral metabolism parameters, including 25(OH)D concentrations. This has led to the suggestion that other pathways are involved in its cardiovascular effects. Thus, FGF-23 may directly influence cardiac remodeling, leading to left ventricular hypertrophy. It has also been reported that it may directly promote cardiomyocyte growth *in vitro*, inducing left ventricular hypertrophy in mice with normal kidney function [27]. Additionally, FGF-23 may act on vascular function through its co-receptor Klotho, which has been shown to regulate endothelial function by increasing nitric oxide availability [28]. It is known that higher FGF-23 concentrations are associated with reduced expression of the co-receptor Klotho, as the cause of endothelial dysfunction [29]. Moreover, FGF-23 has been related to the presence of vascular calcifications. Clinical observational reports have described a positive relationship between FGF-23 concentrations and vascular calcification in patients at different stages of CKD [30–33]. This evidence suggests that FGF-23 would affect vascular calcification directly and/or indirectly (i.e., the effect of FGF-23 on Calcium x Phosphate product regulation, which may predispose to vascular calcification). However, the precise mechanism of such an effect remains unknown. The exact mechanisms responsible for the increase in AS are not fully understood but are likely to reflect complex interaction between structural and functional changes in the arterial wall [34]. Thus, the FGF-23-Klotho axis could increase AS affecting both structural (vascular calcifications) and functional (endothelial function) components of the vascular wall.

Vitamin D deficiency has also been associated with increased cardiovascular disease [3,4] and has been linked to major cardiometabolic risk factors, such as obesity, arterial hypertension or diabetes [35]. Further, vitamin D deficiency is prevalent in patients with T1DM [36–38]. However, previous data evaluating the association between vitamin D levels and cardiovascular disease in T1DM are contradictory. In the CACTI study, vitamin D deficiency (defined as <50nmol/L) was associated with the presence of coronary artery calcium (CAC) (Odds ratio 3.3 (95% CI 1.6–7.0)) [39]. In addition, severe vitamin D deficiency (defined as vitamin D levels equal to or below the lower 10^th^ percentile) independently predicted all-cause mortality in a prospective study with 220 patients with T1DM [5]. However, other authors found no association between vitamin D concentrations and measurements of subclinical atherosclerosis, such as CAC, carotid intima-media thickness or AS [40,41]. In our study, we found that lower serum concentrations of vitamin D were also associated with higher aPWV, even after adjusting for classical cardiovascular risk factors and other potential confounders. The discrepancies between studies may reflect differences in study population, study design or adjusted covariates. Interestingly, our study is the first one to simultaneously report data on vitamin D and FGF-23 blood concentrations in T1DM. Despite the cross-sectional design and the relative small sample size, our data suggest that the inverse relationship between vitamin D levels and AS would not be explained solely by FGF-23, and that vitamin D would play its own role *per se*.

We are aware that the cross-sectional design of the present study cannot preclude causality or temporal ordering of the association among AS, FGF-23 and vitamin D concentrations. In addition, the variation in AS attributed to FGF-23 levels, despite being significant remains a low percentage of this clinical condition. Thus, the observational design does not allow us to ensure complete control of all the potential (unknown) confounding factors, especially those related to mineral metabolism (such as solar exposition or dietary intake of calcium, phosphate and vitamin D). Finally, the lack of associations among AS, FGF-23 and vitamin D in the controls could be because the dispersion of aPWV and FGF-23 concentrations data, which was much lower in controls than in patients with T1DM.

In conclusion, the current study shows for the first time that both higher FGF-23 plasma and lower vitamin D serum concentrations are associated with AS in patients with T1DM and no previous cardiovascular events. Prospective studies are warranted to ascertain the potential pathogenic role of FGF-23 and vitamin D in the development of AS and cardiovascular disease in patients with T1DM.

## Abbreviations

aPWV: aortic pulse wave velocity
AS: arterial stiffness
CAC: coronary artery calcium
CAD: coronary artery disease
CKD: chronic kidney disease
DBP: diastolic blood pressure
ED: endothelial dysfunction
eGFR: estimated glomerular filtration rate
FGF-23: Fibroblast Growth Factor 23
hsCRP: high-sensitivity C-reactive protein
ICAM-1: intercellular adhesion molecule-1
IL-6: interleukin 6
MAP: mean arterial pressure
PTH: parathyroid hormone
SBP: systolic blood pressure
sTNFαR: soluble fraction of tumor necrosis factor α receptor
T1DM: type 1 diabetes mellitus
T2DM: type 2 diabetes mellitus
sVCAM-1: vascular cell adhesion molecule-1
25(OH)D): 25-hydroxy-vitamin D
